# Better preventing and mitigating the effects of Covid-19

**DOI:** 10.2144/fsoa-2020-0051

**Published:** 2020-05-20

**Authors:** Greg Maguire

**Affiliations:** 1 BioRegenerative Sciences Inc., NeoGenesis Inc., San Diego, CA 94704, USA; 2 The California Physiological Society, Berkeley, CA 94704, USA

**Keywords:** Covid-19, diet, exposome, immunity, inflammation, SARS-CoV-2

## Abstract

Currently, there are no proven medical treatments against SARS-CoV-2, the virus responsible for Covid-19. In addition to the all important public health measures needed to prevent the spread of this disease, a number of strategies related to our exposome are recommended herein, to better prevent and mitigate the effects of a SARS-CoV-2 infection through enhancement of our immune system and reduction of inflammation.

## Lay abstract

Viral infections such as that associated with Covid-19 could potentially be mitigated using lifestyle strategies that include a diet rich in whole plants, complete with soluble and insoluble fiber to enhance the immune system and reduce inflammation. In this manner, the probability of being infected may be reduced, and the severity of the disease mitigated. This article discusses potential strategies.

The SARS-CoV-2 virus is currently the focus for many researchers from around the world, given it is so deadly and so little is known about it. An excellent recent review highlights much of what we currently know [1]. Severe acute respiratory syndrome coronavirus 2 (SARS-CoV-2) may have arisen from a genetic shift of coronaviruses, which involved rapid evolution of the virus [2] through the recombination of two viruses within one bat [3]. Bats are important vectors for viruses, including SARS-CoV-2, because bats can carry a high viral load due to mechanisms that they have to protect themselves from the virus; including a high and sometimes constant expression of the antiviral cytokine, IFN-α [4] and a reduction in the resultant inflammasome induced by IFN [5]. The results of the genetic shift(s) in bat viruses have been tragic. We are early into the world outbreak of Covid-19, but already, as of February 2020, Covid-19 has killed more people than severe acute respiratory syndrome (SARS) and Middle East respiratory syndrome (MERS) combined [6].

Hospitals and clinics are notorious for spreading infectious diseases, even in the world’s most prestigious hospitals such as the National Institute of Health hospitals in the USA [7]. The problem is no different in the beautiful and modern town in northern Italia, Bergamo, where physicians have reported that hospitals are the ‘main Covid-19 carriers’ of the SARS-CoV-2 virus [8]. As the world mobilizes to fight this pandemic and develop new therapeutic strategies, including stem cell-based therapeutics [9], passive antibody therapy [10], traditional Persian herbal medicines [11], new nonviral mRNA vaccines [12], nonspecific immune enhancing vaccines such as the BCG vaccine [13] and a number of other potential drugs [14], many questions remain such as: is the virus less transmissible after several passages in humans? [15]. Like SARS-CoV, the SARS-CoV-2 virus is highly transmissible, even in those who are asymptomatic (~86%) [16], and is very stable, remaining viable for hours and even days on some surfaces [17,18], allowing for robust fomite spreading. Therefore, thorough and frequent washing of hands is important to reduce transmission [1]. Some recent (as-yet un-peer-reviewed) models predict that recurrent wintertime outbreaks of SARS-CoV-2 will probably occur after an initial pandemic wave [19]. In the meantime, physicians must consider all possible strategies to prevent and mitigate the effects of Covid-19. Understanding that most diseases are related to our exposome [20] that our health status of our lungs is highly dependent on all of the molecules that we are been exposed to our lifetime [21], and learning lessons from Mother Nature and her bats, here I present a number of strategies for consideration that will enhance our immune systems and reduce inflammation.

## Mitigating Covid-19 through medical procedures

To protect the population, including healthcare workers themselves, symptomatic and asymptomatic patients should remain at home in isolation unless the sequelae progresses to severe symptoms, including difficulty breathing, fever and chest pain [22]. An online assessment tool from the University of Southern California Keck School of Medicine (USC; CA, USA) is available to help determine the severity of infection [23]; however, beyond palliative care, not much can be done for patients in critical condition and requiring ventilation. Use of a high-flow nasal cannula or noninvasive ventilation may not be warranted until the patient has viral clearance of the SARS-CoV-2 virus [24,25] because noninvasive ventilation is unlikely to improve patient outcomes [25], and high oxygen levels may reduce immunity and exacerbate infection [26]. However, prone placement of the patient, combined with high-flow nasal cannulation has been demonstrated to reduce mortality in moderate-to-severe acute respiratory distress syndrome [27]. Vitamin C infusion is unlikely to be useful [28], although the authors of this study found a number of positive effects of high dose, intravenous vitamin C infusion in septic patients [29], a common complication of Covid-19 [30]. Corticoid steroid use in Covid-19 patients reduces immunity and is therefore not recommended [31], unless there is another indication requiring its use. Similarly, bone marrow stem cell transplants are being widely tested for Covid-19 throughout the world but run the risk of reducing immunity through a phenotypic change of T cells to a more aged type [32,33]. While performing these procedures requires more study, they may increase the risk of infection for physicians, nurses, other healthcare workers and other noninfected patients without benefit to the Covid-19 patient.

In a recent study, the intensive care unit mortality rate among Covid-19 patients in China, with required noninvasive ventilation, was 79% (23/29) and reached 86% (19/22) among those who required invasive mechanical ventilation (intubation) [34]. Unfortunately, physicians can do little for most of their Covid-19 patients beyond palliative care or antibiotics for secondary pneumonia infection, where a separate study demonstrated that half of the nonsurvivors experienced a secondary infection, with ventilator-associated pneumonia occurring in 31% (10/32) of patients requiring invasive mechanical ventilation [35]. Nearly all Covid-19 patients have received antibiotics; however, scientists in the UK are warning that antibiotic resistance may become a problem during this pandemic [36]. This may suggest that vaccination for bacterial pneumonia may be beneficial for preventing secondary infections in Covid-19 patients.

On the positive side, studies (as-yet un-peer-reviewed) in Macaque monkeys in China have suggested that once infected with the SARS-CoV-2 virus, immunity develops, and infection does not occur again when inoculated with the virus a second time [37]. While immunity for respiratory coronaviruses that cause the common cold last for about a year, in a Covid-19-related disease, SARS-CoV, SARS-specific antibodies were maintained for an average of 2 years in patients, and a significant reduction of immunoglobulin G-positive percentage and titers occurred in the third year [38].

## Mitigating Covid-19 through diet & exposome

Two immune systems – the innate and the adaptive – operate together in humans, both of which can be modulated by our exposome, including diet, drugs and supplements. The innate immune system is initiated first and automatically and directly attacks invading organisms – this includes macrophages. Meanwhile, the adaptive immune system follows the innate immune system response by ‘learning’ to attack specific invading organisms and remembers them for up to 10 years [39], so that the immune attack can be more specific, and faster during future infections – this includes T cells and B cells [40]. The T cells will have learned to directly attack infected host cells and destroy them, while the antibodies (IgM and IgG) produced by B cells travel in the blood to directly attack the virus itself to disable it [41].

What can you do to become healthier and improve your innate and adaptive immune systems? First, eat vegetables, with soluble and insoluble fiber, limit salt [42] and sugar intake [43], refrain from processed oils – including olive oil – especially coconut oil and even medium-chain triglycerides [44,45]. Studies in human patients led by Ralf Linker at the University of Erlangen (Bavaria, Germany) demonstrated that processed oils shifted T cells to a more proinflammatory state, while short-chain fatty acids (propionic acid and butyrate) were found to do the opposite [45].

Second, exercise regularly. Moderate exercise may be best, although even intense exercise such as running a marathon may be helpful and strengthen the immune system [46]. The mechanisms by which exercise modulates immunity are not well understood. However, considering the importance of T cells in clearing viral infections, exercise may stimulate the production of naive T cells, as previous studies determined that contracting skeletal muscle produces IL-7 [47], which may increase thymic mass and function [48]. In addition, sunlight in moderate amounts enhances your immune system, initially in the skin and then systemically, through the production of vitamin D, which along with other mechanisms enhances T-cell function [49,50].

Third, refrain from drinking alcohol. Alcohol disrupts ciliary function in the upper airways, impairs the function of immune cells, such as alveolar macrophages and neutrophils and weakens the barrier function of the epithelia in the lower airways [51].

More specifically, to mitigate the effects of a viral infection, a mostly plant-based diet that includes significant amounts of insoluble and soluble fiber, may be preferable. Even if one’s traditional diet includes meat, dairy and processed fats, the inclusion of fiber alone can improve adaptive immune cell function [52]. Thus, the strategy proposed here is to bias one’s diet as much as possible toward the following:

- Eat whole-grain carbohydrates because carbohydrates boost your immunity in many ways [53].
- Increase the consumption of soluble fiber as it regulates the innate and adaptive immune systems to better fight viral infection and to better resolve the inflammation induced by the activation of the immune system [54].
- Consume insoluble fiber, as it will upregulate the immune system in a number of ways, including inducing mechanical autophagy that helps to clear infectious agents [55,56].

Eating a predominantly plant-based diet rich in many types of antioxidants sets up an antioxidant cascade [57], which also helps to fight viral infection [58,59]. For example, broccoli sprouts have also been demonstrated to increase the ability of T cells to produce a virus-killing chemical called granzyme B, presumably helping to fight the induced respiratory infection in test subjects who had an induced immune reaction using a live-attenuated influenza vaccination [60]. In another study, blueberry consumption for 6 weeks increased natural killer cell counts, and acute ingestion reduced oxidative stress and increased anti-inflammatory cytokines [61]. Thus, blueberries may help to fight viral infection and reduce the resulting inflammation. In a series of studies, Colin Campbell at Cornell University (NY, USA) determined that a plant-based diet suppressed liver cancers caused by hepatitis B virus infection, suggesting that in addition to inhibiting liver cancer started by the hepatitis B virus, the same nutrition also likely inactivated the virus itself, otherwise, the virus would likely continue to initiate new cancer development [62].

Furthermore, the SARS-CoV-2 virus probably originated in bats and then spread to other animals with which humans interact, including those animals in our food supply. So called ‘wet markets’ world-wide, including in the USA, where live animals are sold have long been known to transmit viral and other disease-causing microorganisms to humans, including respiratory viruses [63]. Packaged meats at the local grocer may do the same. In a study from The Netherlands, scientists found that compared with meat-eating donors, the incidence of hepatitis E virus infection is significantly lower among people not eating meat, indicating that meat consumption is a risk factor for viral hepatitis E virus infection [64]. And recent studies in China found that some coronaviruses have spilled over to pigs [65], meaning that handling pork or eating undercooked pork may be a risk factor for viral infections.

Next, avoid ketogenic diets (high-fat, high-protein, low-carbohydrate) because they will suppress the immune system [66,67]. High-fat consumption of cholesterol, processed oils and saturated fats will decrease immunity, leaving you vulnerable to viral infections [68]. Studies have shown that a diet containing reduced saturated fat and cholesterol led to manipulation of lipid metabolism in monkeys, which in turn provided significant benefits by slowing down simian immunodeficiency virus disease progression [69]. Furthermore, reducing fat intake will decrease serum angiotensin-converting enzyme (ACE) levels [70] and may, therefore, decrease one’s risk factor for Covid-19 by decreasing the number of binding sites for SARS-CoV-2 in our respiratory and pulmonary tissues. Meats, eggs, coconut and palm oil are high in saturated fats and therefore should be avoided. In addition, consuming large volumes of fish, or fish oil, will also lower immunity [71], as fish oil supplements work to reduce inflammation because they reduce immune function. You, especially do not want this during an infection given that T-cell function may be compromised by the supplements [72].

## Mitigating Covid-19 through proper drug use

Nonsteroidal anti-inflammatory drugs, such as Ibuprofen, may also reduce immunity. This is because nonsteroidal anti-inflammatory drugs reduce the production of antibodies [73]. The use of biologics as therapeutic agents for rheumatoid arthritis may increase the probability of Covid-19 infection given that etanercept, infliximab or adalimumab can increase the risk of viral and bacterial infections [74]. Therefore, these patients should use extra precaution, following the strict orders of their physicians and carefully practicing appropriate public health measures, such as quarantining.

The SARS-CoV-2 virus attaches itself to a number of sites in the body using ACE2 [75], including in our alveoli in the lungs [76]. While there is no evidence, research is currently underway to determine whether the upregulation of ACE2 receptors in those cardiovascular patients on ACE inhibitors and angiotensin receptor blockers exacerbates or reduces the Covid-19 infection [77,78]. ACE2 is expressed in numerous immune cell types [79], including the precursor cell to many immune cells, bone marrow stem cells [80]. ACE inhibitors regulate the adaptive and innate immune systems and may decrease immunity to viral infection, although the results are mixed [79]. In view of insufficient research, numerous groups at leading academic medical centers have advised cardiac patients in need of these medications to be treated [81,82]. According to oncologist Richard Schilsky, chief medical officer of the American Society of Clinical Oncology, “different cancers produce immune suppression to different extents”, and according to an article in *The Lancet*, “individuals who are undergoing active chemotherapy or radical radiotherapy for lung cancer, and patients with cancers of the blood or bone marrow may be at particular risk for infection” [83]. Therefore, patients on chemotherapy for cancer should talk to their oncologist about whether the chemotherapy drug being prescribed is beneficial. In the USA, 18 of 36 US FDA-approved cancer drugs using surrogate end points were shown to have no effect on the cancer. These drugs would, therefore, theoretically only do harm, while potentially increasing the probability of a Covid-19 infection [84].

## Conclusion

As we await the development of antiviral medications and a SARS-CoV-2 vaccine, public health measures are the best means to fight the Covid-19 pandemic. However, careful attention to one’s exposome, including not only exposure to infectious agents but also diet and medications, may be an important strategy to help enhance the immune system response to SARS-CoV-2 and, also mitigate immune system dysfunction, including exacerbated inflammation.

## Future perspective

While humans continue to genetically and epigenetically coadapt with our planet, increasing population, rapid and extensive interactions with fellow humans and other organisms, including zoonotic viruses, means that adaptation in humans will not occur as rapidly as that of other biological entities, such as some viruses, which can internally coevolve their subunits in few passages [85]. Nor will humans adapt rapidly enough to remain healthy in an ever changing and expanding exposome, including the newly made more than 2000 chemicals created every year [86]. To better live with the rapidly changing environment being created, new therapeutic strategies must emerge, for example, using systems therapeutics for physiological renormalization [87,88] to bring our bodies into a state of allostasis [89], where the immune system components are in balance to fight infection, and inflammation can be properly resolved. An important example of renormalizing the immune system is in cancer therapeutics where ‘checkpoint inhibitors’ renormalize T cells to attack the tumor, a technology for which the Nobel Prize in Physiology or Medicine was awarded to James Allison [90]. When fiber is ingested by the patient being dosed with checkpoint inhibitors, the therapeutic effect is enhanced by the fiber, better enabling T-cell function [52] – demonstration of a system therapeutic approach to physiological renormalization. Most important to man’s future, current public health measures and organizations must be maintained, such as support for the WHO and its programs throughout the world. Better public health measures must also be enacted, such as those proposed steps needed for cooperative scientific programs between the USA and China to better protect the global community from zoonotic diseases, such as Covid-19 and future emerging diseases [91]. Unfortunately, ‘exogenous shocks can increase the significance of nationalism’ [92]. Thus, the global Covid-19 pandemic, an exogenous shock, may be bringing forth the articulation of nationalism among some world leaders [93], just at a time when cooperation throughout the world is needed. Not only will support of the WHO lead to better public health measures and scientific progress, but proposed scientific programs across countries [91], such as that recently presented by The Human Cell Atlas Lung Biological Network [94], will increase the rate of scientific progress in understanding these diseases. This group of scientists from around the world found significantly enhanced ACE2 expression as we age. Data such as these may help to explain why children have a reduced probability of Covid-19 infection [95], and may help to stratify the population into those at high risk for infection and therefore develop appropriate public health measures to protect such groups.

Furthermore, new technologies such as artificial intelligence (AI) have proven valuable for early warning once an outbreak has occurred. As an example, The Global Public Health Intelligence Network [96], an AI-based surveillance system initiated and run by Health Canada and the WHO, currently analyzes more than 20,000 online news reports in nine languages daily. This AI system was credited with the first alerts for SARS and MERS [96]. If these big data from AI are supported by epidemiological outbreak investigations on the ground, then false alarms can be avoided, and proper public health strategies developed. Strategies should include proper diet for those at risk to better prevent and mitigate infectious diseases. Therefore, AI surveillance [96] and outbreak investigations, such as that used to predict outbreaks of the dengue viral infection [97], along with proper public health measures need to be scaled up and integrated into a comprehensive global early warning system and follow-on public health measures. These follow-on public health measures, in order to be more effective, should include careful consideration of the exposome and nutritional needs to better prevent and mitigate infectious diseases. In the near future, medicine needs to perform more autopsies on Covid-19 patients, as autopsy is a key means to learn about diseases [98], and recent autopsies of Covid-19 patients have found, for example, complement-associated microvascular injury and thrombosis with resultant stroke [99].

**Executive summary**

- Emerging infectious diseases have been and will continue to be threats throughout the world.
- Currently, no effective medical interventions are approved for Covid-19.
- Public health measures are currently the most effective means to control Covid-19 infection.
- Controlling one’s exposome, including diet, may be one important means to limit infection and control the ensuing inflammation and immune system dysfunction elicited by the SARS-CoV-2 virus.
- Inclusion of a rich variety of fruits, vegetables, fiber and whole grains that enhances immune function and helps to resolve inflammation holds potential as a strategy to better disease prevention and outcomes during the Covid-19 pandemic.
- Scientific, medical and public health knowledge must be coalesced from around the world using formal plans [89], programs [94,95] and organizations (e.g., WHO) to develop and bring forth the knowledge about Covid-19, so that this and other coming disease pandemics can be effectively prevented or mitigated.
